# Applying a cumulative risk framework to drinking water assessment: a commentary

**DOI:** 10.1186/s12940-019-0475-5

**Published:** 2019-04-30

**Authors:** Tasha Stoiber, Alexis Temkin, David Andrews, Chris Campbell, Olga V. Naidenko

**Affiliations:** Environmental Working Group, 1436 U Street NW Suite 100, Washington, DC 20009 USA

**Keywords:** Drinking water, Cumulative risk, Disability weighting, Toxicity assessment

## Abstract

The health risks of drinking water contaminants and the economic benefits of drinking water standards are typically assessed one chemical at a time, an approach that misses the health impacts of co-occurring contaminants in drinking water. In contrast, a cumulative risk framework has become common in air quality evaluations such as the U.S. Environmental Protection Agency’s National Air Toxics Assessment. We posit that the drinking water field would benefit from making the transition to a unified assessment framework for multiple contaminants that can overcome the long-standing challenge of treating cancer and non-cancer contaminants separately. Here we present a cumulative risk methodology that combines a risk-based cancer metric with a weighted health indicator index for non-cancer contaminants and incorporates disability weights from the Global Burden of Disease study. Our methodology generates a numeric toxicity score reflecting the potential health impacts for the sum of contaminants present in each sample of drinking water. Further research is needed to refine the risk and toxicity parameters for specific contaminants and to address the mode of interaction between co-occurring chemicals. As this cumulative risk model goes through future refinements, we anticipate that it would provide information that can help communities and policy makers evaluate different options for drinking water treatment.

**Electronic supplementary material:**

The online version of this article (10.1186/s12940-019-0475-5) contains supplementary material, which is available to authorized users.

## Background

Over the past two decades, exposure research has documented the co-occurrence of numerous contaminants in drinking water, yet the drinking water policy framework has not yet caught up with this practical reality. With some exceptions, such as the treatment of drinking water disinfection byproducts as a group, most water contaminants are typically assessed one chemical at a time. This approach creates two problems. First, scientists and policy makers lack information about the full scope of health impacts from multiple contaminants in drinking water. Second, economic impact analyses of contaminant reduction strategies for single chemicals likely undercount the benefits that could come from the removal of multiple contaminants.

As advocated by the groundbreaking “Science and Decisions” report from the National Research Council, published in 2009, “cumulative risk assessment would be most valuable to both communities and decision-makers when it can provide information about the health implications of alternative control options” [1]. We envision that future applications of the cumulative assessment framework would help advance water treatment strategies for multiple co-occurring contaminants and thus help protect public health. The purpose of this commentary is to bring together cumulative risk methodologies established in air quality studies and published literature on water quality assessment, as well as to stimulate much-needed research on cumulative risk in the drinking water field.

## Main text

### Implementing cumulative risk assessment: what drinking water researchers can learn from air quality assessment methodologies

Cumulative risk assessment for drinking water has lagged behind similar methodologies already standard in air quality evaluations. In our estimate, the slow adoption of cumulative methods in drinking water assessments is at least partly due to the variety of health outcomes caused by drinking water contaminants. While acknowledging the scientific challenges of assessing the impacts of co-occurring chemicals on multiple body systems, we believe that the drinking water field can start with the application of existing cumulative risk methodologies established for air quality. Moreover, high-quality data on contaminant occurrence collected via required annual testing for community water systems in the U.S. are increasingly available in electronic format, which facilitates innovative research on co-occurring contaminants.

The fundamental question that bedevils cumulative risk assessment is the overall health impact of contaminant mixtures. Theoretically, additive, synergistic and antagonistic interactions are possible for mixtures of contaminants that affect even a single organ or tissue [1]. The 1996 amendments to the Safe Drinking Water Act required the U.S. Environmental Protection Agency (EPA) to conduct research to “develop new approaches to the study of complex mixtures, such as mixtures found in drinking water, especially to determine the prospects for synergistic or antagonistic interactions that may affect the shape of the dose-response relationship of the individual chemicals and microbes” (Public Law No. 104–182, 104th Congress). Yet for drinking water contaminants that target diverse body systems – for example, reproductive, endocrine, neurodevelopmental, dermal, and respiratory – and those associated with cancer toxicity outcomes, the question of chemical interactions seemingly becomes intractable at the present state of scientific research.

In the initial stage, a cumulative framework can start with a simple additive approach as a basis for the future development of more sophisticated, data-driven risk models. Additive risk methodologies have been effectively used for the cumulative evaluation of air quality within the EPA’s National Air Toxics Assessment, first developed in the 1990s [2]. In the EPA’s approach, the overall cancer risk metric represents a statistical probability of developing cancer over a lifetime of exposure to an individual carcinogenic contaminant or a mixture of contaminants at specified levels. A risk of 10^− 6^ corresponds to a contaminant concentration that, upon a lifetime exposure, would cause one cancer case in a population of 1 million people, which is sometimes described as an acceptable *de minimus* risk. The cumulative cancer risk estimates are aggregated via a simple addition of cancer risk levels for individual contaminants [2]. These cumulative values are commonly presented as a 10^− 6^, 10^− 5^, or 10^− 4^ and express the lifetime risk of developing 1 case of cancer in a population of 1 million, 100,000 or 10,000 people, respectively [2].

The EPA’s technical support materials for the National Air Toxics Assessment note that the true value of the cumulative risk is not known and that the actual risks could be lower than predicted [2]. These risks could also be higher, for example, due to potential synergistic interaction among co-occurring carcinogens. As more precise information on chemical interactions becomes available, this initial model would become more refined and reliable. Meanwhile, an additive approach to cumulative cancer risk assessment can help scientists and risk managers carry out an overall risk characterization.

### Cumulative lifetime cancer risk from drinking water contaminants

Applying the cumulative cancer risk framework to the 2011–2015 drinking water dataset for community water systems in California, we calculated that up to 15,449 lifetime cancer cases could be related to drinking water quality across the state (Table 1). Our assessment is based on water quality data published by the California State Water Resources Control Board, and the data collected under the EPA’s Unregulated Contaminant Monitoring Rule. Both datasets are publicly available online and can be freely downloaded.

**Table 1:** Cumulative cancer risks from drinking water contaminants in community water systems in California

Cumulative cancer risk	Number of community water systems	Exposed population	Estimated number of lifetime cancer cases	Percent contribution to total drinking water-related lifetime cancer cases
> 10^−3^	495	3,103,996	4860	31%
10^−4^ – 10^−3^	1177	28,497,278	10,427	68%
10^−5^ – 10^−4^	435	2,598,480	149	1%
< 10^−5^	107	4,510,325	13	0.09%
Total		38,710,079	15,449	

Details on the drinking water dataset analyzed here, all contaminants included in this study, and formulas for calculating cumulative risk are listed in the Additional files 1 and 2. Overall, we analyzed water quality profiles for 2737 California community water systems, which are defined by the EPA as systems that supply tap water to the same population year-round. Since approximately 98% of California residents rely on public water systems [3], our dataset offers a comprehensive reflection of the state’s drinking water quality.

**Fig. 1:**
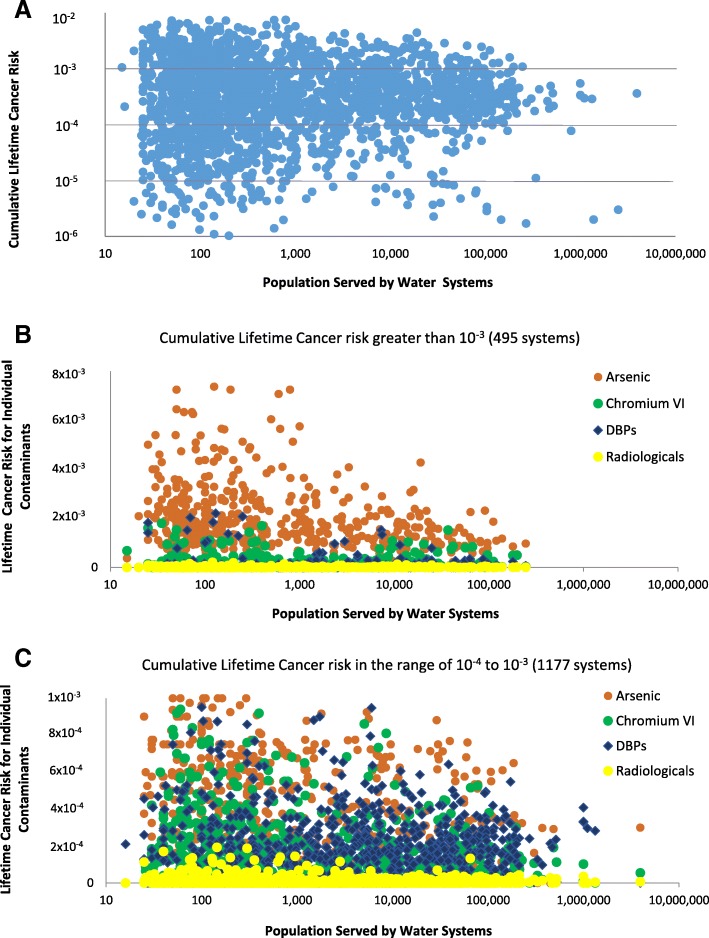
**a.** Distribution of cumulative lifetime cancer risks for California public water systems. Y axis: cumulative cancer risk for each water system; X axis: water utilities plotted according to the size of population served**. b** and **c**. Contribution to the lifetime cancer risk by individual contaminants or contaminant groups. DBPs refer to nine carcinogenic disinfection byproducts listed in Additional file 1: Table S1. **b**: Public water systems with cumulative lifetime cancer risk greater than 10^−3^. **c**: Public water systems with cumulative lifetime cancer risk between 10^− 4^ and 10^− 3^

As Table 1 and Fig. 1a demonstrate, water systems with the highest risk serve predominantly smaller communities, with population under 10,000 people. 495 public water systems in California, serving a population of approximately 3.1 million, contained cancer-causing contaminants posing a cumulative lifetime cancer risk greater than 1 × 10^− 3^, resulting in 4860 estimated lifetime cancer cases. Approximately 28.5 million people in California depend on water systems with cumulative lifetime cancer risks in the range of 10^− 4^ to 10^− 3^, resulting in 10,427 estimated lifetime cancer cases.

A graphical analysis of water-system-level lifetime cancer risks highlights the contribution of individual contaminants and contaminants groups to the cumulative risk profile. As other researchers have reported [4], arsenic is a major contributor to cancer risk in smaller water systems in California and in the highest cancer risk tier (Fig. 1b), whereas other contaminants, such as disinfection byproducts and hexavalent chromium, become important risk contributors in the second highest risk tier (Fig. 1c). Across the state, 47% of estimated lifetime cancer cases is due to arsenic pollution in drinking water (Additional file 1: Table S1), whereas the rest are due to the group of nine carcinogenic disinfection byproducts (34%); hexavalent chromium (16%); radioactive elements (2%); and carcinogenic volatile organic chemicals (1%).

We calculated the annual cancer risk related to drinking water contaminants by dividing the 15,449 estimated lifetime cancer cases by 70 years, the typical lifetime. This results in an estimated number of 221 annual cancer cases due to drinking water contaminants in California, a state with approximately 39 million residents. Our estimate for California is similar to the findings of a 2017 study by DeFelice [5] that reported a mean of 54 annual cases (range: 28–79 cases) attributable to regulated drinking water contaminants in North Carolina, a state with 10 million residents. The concordance between our analysis and previously published results points to the robustness and scientific promise of the cumulative cancer risk methodology for drinking water quality assessment.

Our research also identified the most concerning water quality scenarios, with estimated lifetime cancer risk greater than 4 × 10^− 3^. Such high risks are found for 43 public water systems in California that serve communities of less than a thousand residents and one water system serving a population of 19,000. This finding reflects a significant environmental justice problem, with water systems serving smaller communities often most in need of resources and infrastructure for safe drinking water, as reported by other studies [3, 4].

It is worth noting that the lifetime cumulative cancer risks calculated here can be considered conservative estimates, because water systems can classify as “non-detects” the test results for contaminants detected at a concentration below the state’s official detection limit for the purposes of reporting and compliance assessment. For several common carcinogenic water contaminants, including arsenic, hexavalent chromium, radium, uranium and tetrachloroethylene, the 10^− 6^ cancer risk level is below the official reporting limit for water utility tests [3]. Thus, it is possible that additional cancer cases could be due to the presence of water contaminants reported as “non-detects” by the drinking water providers.

### Using the global burden of disease framework and a relative health Indicator metric to integrate cancer and non-cancer assessment

Although federal and state contaminant assessments have traditionally addressed cancer and non-cancer risks independently, this separation between health outcomes stands as an outdated legacy of the environmental regulations’ development in the U.S. that does not serve the goal of public health protection in the twenty-first century. We believe that treating all co-occurring contaminants in a unified framework would help advance ground-breaking strategies for pollution prevention. We also note that in the Global Burden of Disease framework [6] and numerous studies that have grown from this innovative methodology, all stressors and exposures are viewed through the single lens of their impact on the overall quality and duration of life, without making a distinction between cancer outcomes and non-cancer outcomes.

A recent research study conducted under the auspices of the Water Research Foundation, a not-for-profit research cooperative governed by water utilities, proposed a Relative Health Indicator metric for assessing the risks from co-occurring contaminants based on the exposure data and information about the health impact of individual contaminants [7, 8]. This metric combines a risk-based cancer index with a hazard index for non-cancer contaminants and weighs their impacts through contaminant severity factors derived from the Global Burden of Disease study. The hazard index approach for non-cancer contaminants is also used in the EPA National Air Toxics Assessment and other EPA programs, whereby non-cancer contaminant impacts are assessed by comparing the exposure with a health benchmark, also called a reference concentration. In the EPA definition, a reference concentration represents a level of a non-carcinogenic contaminant at which adverse health effects are assumed to be unlikely [2]. A ratio between exposure and a reference concentration produces a hazard quotient, which is a useful but limited metric, because it does not inform the risk manager about the probability of an adverse effect following an exposure event.

Seidel [7] and Alfredo [8] address this difficulty by including two additional parameters into their cumulative Relative Health Indicator metric. The first parameter is the contaminant severity factors derived from the Global Burden of Disease disability weights for specific health sequelae. The second parameter is the incidence factor that, in theory, should reflect the probability of an adverse health outcome following exposure to a specific contaminant. The original Water Research Foundation study [7] used a fixed incidence factor of 1%, which implies that when exposed to the given concentration of the contaminant, 1% of the population is likely to experience the related health effects [7]. Here we used the same default factor, and we discuss the impact of the incidence factor on the overall cumulative score further in this commentary.

With the Relative Health Indicator metric, we analyzed the California water quality dataset to probe and illustrate the scientific underpinnings of this model and to highlight the parameters that should be refined through further research. For this exploratory analysis, we started with the cumulative cancer risk estimates described above and included exposure and toxicity data for the four most common non-cancer contaminants in drinking water in California. Water in specific communities may contain contaminants other than the ones included in this study. The framework presented here can be adapted to incorporate any contaminants for which health benchmarks are available, and thus extended for chemicals not included in this overview.

Methodological details for our application of the Relative Health Indicator model are listed in Additional file 2. Briefly, this process involves the identification of critical health effects associated with exposure to a contaminant; mapping of the critical health effects to the closest disease or health condition within the latest Global Burden of Disease framework [6]; and finally, selection of a corresponding disability weight as the contaminant severity factor corresponding to the critical health effect. Severity factors for carcinogenic contaminants were identified from peer-reviewed research (Additional file 1: Table S2). The impact of different cancers on the length and quality of life after diagnosis varies significantly; this is reflected in a broad range of disability weights for various cancer types and stages analyzed by the Global Burden of Disease studies. In this analysis, we used the cancer severity factors corresponding to the diagnosis and initial treatment stage. Additional file 1: Table S3 lists the non-cancer severity factors for chlorate, manganese, nitrate and vanadium, as well as for the non-cancer effects of arsenic and hexavalent chromium.

**Fig. 2:**
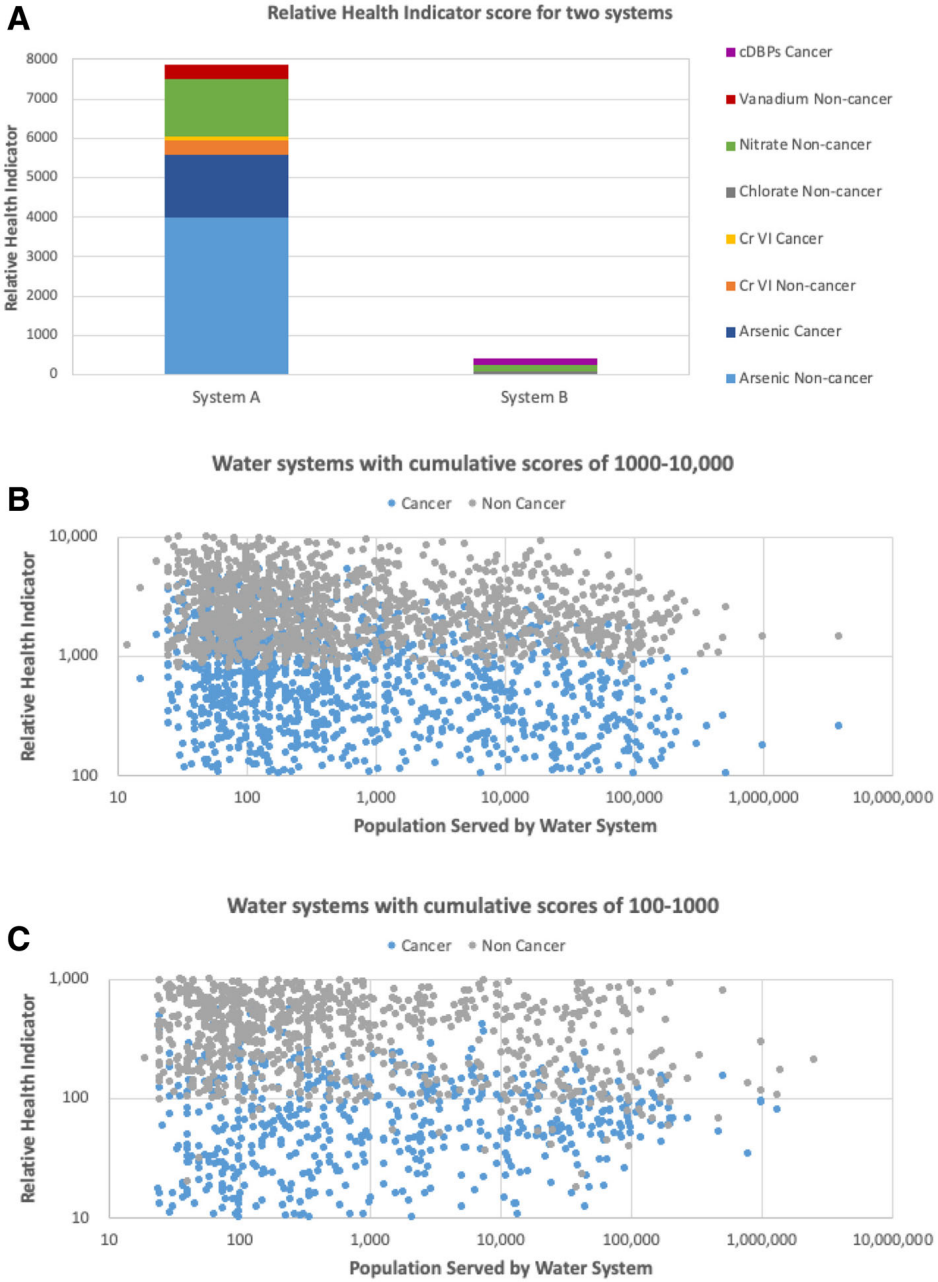
**a:** Relative Health Index scores for water quality scenarios for two California water systems serving 70,000 to 90,000 people. cDBPs refer to nine carcinogenic disinfection byproducts listed in Additional file 1: Table S1. **b** and **c**: Distribution of cumulative Relative Health Indicator scores for cancer and non-cancer effects in the highest risk tier (**b**, cumulative scores greater than 1000) and the second highest risk tier (**c**, cumulative scores of 100–1000). For all data presented in this figure, severity score of 0.041 was used for arsenic as is the most conservative estimate of arsenic’s non-cancer toxicity (Additional file 1: Table S3); and incidence factor of 1% was used for the calculation of all non-cancer scores

Cumulative cancer and non-cancer Relative Health Indicator scores for community water systems in California offer a thought-provoking illustration of a state-level water quality assessment. Figure 2a shows two specific scenarios for different water quality profiles, demonstrating the contributions of individual contaminants to the cumulative scores. Unlike the cumulative cancer risks metrics which reflect the probability of cancer development on a population basis, the Relative Health Indicator scores convey information about the overall health impacts due to the presence of multiple contaminants but do not represent the probability of disease. At this stage of model development, the Relative Health Indicator scores are most useful for a relative comparison between different water quality scenarios. For ease of visualization, scores are presented on a numerical scale, and all formulas for calculating these scores are listed in Additional file 2.

Figure 2b and c present cancer and non-cancer scores for the entire state of California. As for the cumulative risk of cancer, the highest Relative Health Indicator scores are observed in smaller water systems. The score distribution conveys the notable contribution of non-cancer effects to the overall score, information that should be considered by drinking water policy makers and communities as they explore contaminant reduction strategies and options for pollution prevention.

### Uncertainties and future research needed for refining a cumulative risk methodology

The scientific uncertainties and limitations associated with deriving data-based incidence factors and with the assignment of contaminant severity factors deserve a detailed investigation of their own. Here we summarize some of these uncertainties and the research needed to address them.

As detailed in a recent commentary by Bellinger, the Global Burden of Disease focuses on “high risk” exposures and health losses and does not zoom in on the subtle and sometimes subclinical effects of environmental contaminants [9]. The same commentary also questioned whether the disability weights assigned to the most common sequelae can gauge correctly the impact of environmental contaminants on population and individual health.

We also note the limitations associated with a default incidence factor for non-cancer health effects. The original Water Research Foundation study suggested a fixed incidence factor of 1% applicable to contaminant effects at any exposure [7], and this default parameter is the source of the greatest uncertainty in the application of the Relative Health Indicator metric. Understandably, the use of a greater incidence factor shifts the cumulative non-cancer scores upward and increases the contribution of the non-cancer score in the overall cumulative score for a given water quality scenario. The premise of a constant incidence factor at different exposure levels is inconsistent with the basic premise of toxicology – “the dose makes the poison” – and with the findings of epidemiological studies that show elevated risk at greater exposures. Yet, like Seidel [7] and Alfredo [8], we recognize a need for starting with a default variable, rather than waiting for decades until more epidemiological data become available.

To probe the impact of varying model parameters on the Relative Health Indicator scores, we examined the cancer and non-cancer effects of exposure to arsenic. In addition to increasing the risk of lung and bladder cancers, arsenic increases the risk of heart attacks, stroke, diabetes mellitus and hypertension [10]. In a review of the possible health sequelae from the Global Burden of Disease framework [6], we identified three disease states related to heart failure, which range from mild to severe and correspond to disability weights of 0.041, 0.072, and 0.179 (Additional file 1: Table S3). Future research may answer the question of which severity factor is most appropriate for arsenic.

**Fig. 3:**
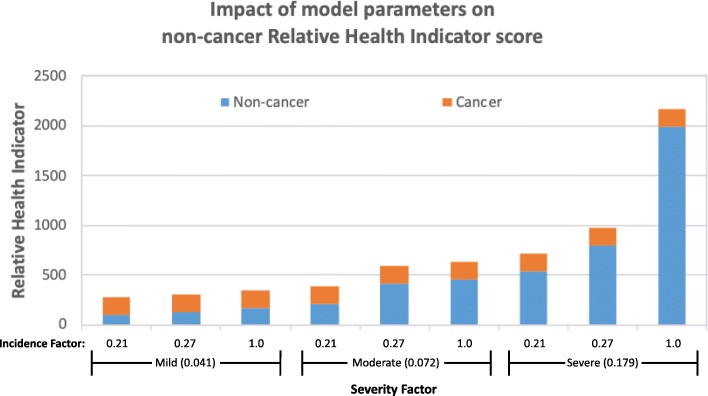
Distribution of Relative Health Indicator scores for three different non-cancer severity factors and three different incidence factors for arsenic. All calculations are conducted for a single arsenic concentration of 1 μg/L. This concentration corresponds to cancer risk level of 2.5 × 10^− 4^, and a cancer Relative Health Indicator score of 180

For an illustration of how the severity and the incidence factors influence the non-cancer Relative Health Indicator score, Fig. 3 presents different scores corresponding to the non-cancer effects due to exposure to arsenic in water at 1 μg/L concentration. The calculations were conducted for three different severity scores (0.041, 0.072, and 0.0179) and three different incidence factors. We included the incidence factor of 1% proposed by Seidel [7] as well as two incidence factors, of 0.21 and 0.27%, derived from an epidemiological study of mortality rate for cardiovascular disease related to arsenic exposure [10], which was between 214 and 271 per 100,000 person years. These nine options produce a range of cumulative Relative Health Indicator scores between 276 and 2169. Notably, the cancer score does not change in this example, as it is calculated based on a published 1 × 10^− 6^ cancer risk level for arsenic (Additional file 1: Table S1).

Depending on the input factors, the non-cancer risk contribution in the overall score ranges from 35 to 92%. For comparison, using the Relative Health Indicator metric with a single set of model parameters for arsenic, Alfredo [8] reported that the non-cancer effects of arsenic constitute approximately 60% of the cumulative score. The parameters used for our analyses in Fig. 2 result in a 72% contribution of arsenic’s non-cancer effects within the cumulative arsenic score.

Notably, the question of the relative risk of heart attacks versus the relative risk of cancer due to arsenic can be answered experimentally through existing and ongoing epidemiologic studies, with current evidence pointing to a higher risk associated with non-cancer outcomes. Yet, similar studies are uncommon for other water contaminants, making it necessary to conduct further research on the appropriate model parameters that can be used for refining the Relative Health Indicator methodology.

## Conclusions

Cumulative cancer risk estimates for drinking water contaminants can be calculated by applying the methodologies already established for air pollutant assessments. Moreover, despite the lingering scientific questions about how to evaluate cancer and non-cancer risks concurrently, we suggest that the Relative Health Indicator framework holds great promise as an assessment and planning tool for the drinking water field. As for other cumulative risk approaches proposed in the literature, there are various uncertainties within our methodology. Some could result in an underestimated risk, and others could result in an overestimated risk. The goal of this commentary is to stimulate a discussion of cumulative risk assessment for drinking water as well as promote scientific research that would help fill in the missing data for individual model parameters. With further refinements, this metric can be used by communities, government agencies and other stakeholders for prioritizing resources dedicated to drinking water treatment and for evaluations of the health and economic benefits of water treatment technologies that reduce multiple contaminants simultaneously.

## Additional files

Additional file 1: **Table S1.** Cumulative cancer risks for drinking water contaminants whose arithmetic mean concentration exceeded the one-in-a-million risk level in more than 20 community water systems in California during 2010 to 2015. **Table S2.** Cancer severity factors at the diagnosis and initial treatment stage, adapted from Soerjomataram et al. (2012). **Table S3** Non-cancer severity factors for common tap water contaminants based on the 2017 Global Burden of Disease study disability weights. (DOCX 27 kb)

Additional file 2: Cumulative relative health indicator formulas, adapted with modifications from Alfredo et al. (2017). (DOCX 16 kb)

## Abbreviations

EPA: U.S. Environmental Protection Agency
IRIS: Integrated Risk Information System
OEHHA: California Office of Environmental Health Hazard Assessment
RHI: Relative Health Indicator
UCMR3: Third Unregulated Contaminant Monitoring Rule
VOCs: Volatile organic chemicals
