# Supplementary material for: Applying a cumulative risk framework to drinking water assessment: a commentary
Source: Environ Health. 2019 Apr 30;18:37. doi: 10.1186/s12940-019-0475-5 (PMC6489338; doi:10.1186/s12940-019-0475-5)
Supplement: Supplementary file 1 — Table S1. Cumulative cancer risks for drinking water contaminants whose arithmetic mean concentration exceeded the one-in-a-million risk level in more than 20 community water systems in California during 2010 to 2015. Table S2. Cancer severity factors at the diagnosis and initial treatment stage, adapted from Soerjomataram et al. (2012). Table S3 Non-cancer severity factors for common tap water contaminants based on the 2017 Global Burden of Disease study disability weights. (DOCX 27 kb) [file 12940_2019_475_MOESM1_ESM.docx]

**Additional File 1**

**Table S1.** Cumulative cancer risks for drinking water contaminants whose arithmetic mean concentration exceeded the one-in-a-million risk level in more than 20 community water systems in California during 2010 to 2015.

| **Contaminant** | **Drinking water concentration corresponding to 10^-6^ lifetime cancer risk**  **(1)** | **Number of public water systems with contaminant levels exceeding the 10^-6^ cancer risk**  **(2)** | **Population exposed to contaminant at levels exceeding the 10^-6^ cancer risk level**  **(3)** | **Estimated number of lifetime cancer cases for California**  **(4)** | **Agency defining 10^-6^ lifetime cancer risk level** | **Year published** |
| --- | --- | --- | --- | --- | --- | --- |
| Arsenic | 0.004 μg/L | 959 | 15,003,196 | 7251 | California OEHHA | 2004 |
| Hexavalent chromium | 0.02 μg/L | 1250 | 32,947,698 | 2448 | California OEHHA | 2011 |
| **Disinfection byproducts (DBPs)** | Not Available |  |  | SUM: 5244 |  |  |
| Bromodichloro-methane | 0.06 μg/L | 845 | 24,331,056 | 2385 | California OEHHA | 2018 (proposed) |
| Dibromochloro-methane | 0.1 μg/L | 821 | 23,802,319 | 1620 | California OEHHA | 2018 (proposed) |
| Chloroform | 0.4 μg/L | 713 | 22,386,686 | 564 | California OEHHA | 2018 (proposed) |
| Bromoform | 0.5 μg/L | 678 | 20,501,727 | 187 | California OEHHA | 2018 (proposed) |
| Trichloroacetic acid | 0.5 μg/L | 486 | 18,066,035 | 178 | U.S. EPA IRIS | 2011 |
| Dichloroacetic acid | 0.7 μg/L | 550 | 19,275,893 | 164 | U.S. EPA IRIS | 2003 |
| Bromate | 0.1 μg/L | 22 | 4,279,718 | 146 | California OEHHA | 2009 |
| **Radioactive elements** | Not Available |  |  | SUM:345 |  |  |
| Sum of uranium-234, uranium-235 and uranium-238 | 0.43 pCi/L | 763 | 19,201,427 | 162 | California OEHHA | 2001 |
| Radium-228 | 0.019 pCi/L | 225 | 5,967,996 | 154 | California OEHHA | 2006 |
| Radium-226 | 0.05 pCi/L | 99 | 6,611,524 | 29 | California OEHHA | 2006 |
| **Carcinogenic VOCs** | Not Available |  |  | SUM:161 |  |  |
| 1,2-Dibromo-3-chloropropane | 0.0017 μg/L | 103 | 2,663,104 | 66 | California OEHHA | 1999 |
| 1,2,3-Trichloropropane | 0.0007 μg/L | 62 | 2,548,031 | 40 | California OEHHA | 2009 |
| Tetrachloroethylene | 0.06 μg/L | 88 | 3,590,968 | 37 | California OEHHA | 2001 |
| 1,4-Dioxane | 0.35 μg/L | 49 | 2,450,876 | 11 | U.S. EPA IRIS | 2013 |
| Trichloroethylene | 0.5 μg/L | 25 | 1,266,183 | 7 | U.S. EPA IRIS | 2011 |
| **TOTAL** |  |  |  | 15,449 |  |  |

1. Drinking water concentration corresponding to 10^-6^ lifetime cancer risk obtained from the websites of the California Office of Environmental Health Hazard Assessment (<https://oehha.ca.gov/>) and the U.S. Environmental Protection Agency Integrated Risk Information System (<https://www.epa.gov/iris>).

2. Arithmetic means for contaminant concentration for each individual water utility were calculated for all available test results for a contaminant within the time frame analyzed. For federally regulated water contaminants that are monitored annually, we used the 2015 data; for contaminants monitored once in several years, we used a longer time frame: 2010-2015 for radiological contaminants and 2013-2015 for arsenic; and, for unregulated contaminants monitored in the UCMR3 program we used the entire data range of 2013 to 2015. Test results reported as “non-detects” were assigned a value of zero and included in the overall data array for the calculation of averages.

3. Sum of population served by utilities with contaminant levels exceeding the 10^-6^ benchmark. Statistics on population served by individual utilities obtained from California SDWIS Safe Drinking Water Information System (<https://sdwis.waterboards.ca.gov/PDWW/>). Adding the total population served listed for the systems in our dataset we found that overall population data overestimated by 12 % the number of California residents served by public water systems [7]. A 12% population correction factor was applied to estimates of exposed population in this Table and elsewhere in the manuscript.

4. Estimated number of contaminant-specific lifetime cancer cases for the exposed population based on formulas below. For contaminant groups, the number represents a summation of estimated number of cases for individual contaminants within the group.

*Cumulative Cancer Risk Formulas*

Lifetime cancer risk for a contaminant = [Exposure] / [Contaminant concentration corresponding to 10^-6^ lifetime cancer risk]

Estimated number of lifetime cancer cases for a water system, a contaminant or for the entire state = Σ [lifetime cancer risk for a contaminant or group of contaminants] x [population served by the water system or systems]

**Table S2.** Cancer severity factors at the diagnosis and initial treatment stage, adapted from Soerjomataram et al (2012).

| **Contaminants, in alphabetical order** | **Primary cancer associated with exposure to the contaminant** | **Cancer severity factor** |
| --- | --- | --- |
| Arsenic | Lung cancer | 0.72 |
| Hexavalent chromium | Stomach cancer | 0.53 |
| Disinfection byproducts | Bladder cancer | 0.27 |
| Other carcinogenic contaminants | Variety of cancers | 0.43 (median for 27 different cancers) |

*Data source*: Soerjomataram I, Lortet-Tieulent J, Ferlay J, Forman D, Mathers C, Parkin DM, Bray F. Estimating and validating disability-adjusted life years at the global level: a methodological framework for cancer. BMC Med Res Methodol. 2012;12:125.

**Table S3.** Non-cancer severity factors for common tap water contaminants based on the 2017 Global Burden of Disease study disability weights.

| **Contaminants, in alphabetical order ^1^** | **Non-cancer health benchmark** | **Reference and year for the health benchmark** | **Critical health effect on which the health benchmark was based** | **Corresponding health condition identified in the Global Burden of Disease Study (2017)** | **Severity factor^2^** |
| --- | --- | --- | --- | --- | --- |
| Arsenic | 0.9 μg/L | California OEHHA (2004) | Vascular disease, including cerebrovascular disease, ischemic heart disease mortality, and hypertension | **Outcome 1**: " Asymptomatic and mild heart failure due to endocrine, metabolic, blood, and immune disorders" | **Outcome 1:** 0.041 |
|  |  |  |  | **Outcome 2**: " Moderate heart failure due to endocrine, metabolic, blood, and immune disorders" | **Outcome 2:** 0.072 |
|  |  |  |  | **Outcome 3**: "Severe heart failure due to ischemic heart disease" | **Outcome 3:** 0.179 |
| Chlorate | 210 μg/L | U.S. EPA UCMR3 (2012) | Thyroid hypertrophy and mineralization | "Mild endocrine, metabolic, blood, and immune disorders" | 0.019 |
| Hexavalent chromium | 2 μg/L | California OEHHA (2011) | Mild, chronic inflammation and fatty changes of the liver | "Mild endocrine, metabolic, blood, and immune disorders" | 0.019 |
| Manganese | 100 μg/L | Minnesota Department of Health guideline for bottle-fed babies (2012) | Neurological effects such as learning and behavior problems | “Symptomatic attention-deficit/hyperactivity disorder" | 0.045 |
| Nitrate | 1000 μg/L | Peer-reviewed study (2017)**^3^** | Increased rate of very preterm birth and very low birth weight | “Mild motor plus cognitive impairments due to neonatal preterm birth complications 32-36wks” | 0.031 |
| Vanadium | 21 μg/L | U.S. EPA UCMR3 (2012) | Altered renal function, increased urea levels in blood plasma and histological changes in kidneys | "Mild endocrine, metabolic, blood, and immune disorders" | 0.019 |

1. Number of California public water systems where 2010-2015 mean contaminant concentrations were greater than the non-cancer health benchmark level identified in this table: Arsenic: 839 systems; Chlorate: 77 systems; Cr VI: 513 systems; Manganese: 252 systems; Nitrate: 1,225 systems; Vanadium: 84 systems.

2. Severity score of 0.041 for arsenic was used in the calcualtions of the Relative Health Indicator scores for California water systems (Figure 2), as is the most conservative estimate of arsenic’s non-cancer toxicity.

3. Stayner LT, Almberg K, Jones R, Graber J, Pedersen M, Turyk M. Atrazine and nitrate in drinking water and the risk of preterm delivery and low birth weight in four Midwestern states. Environ Res. 2017;152: 294-303.
