# Supplementary material for: Applying a cumulative risk framework to drinking water assessment: a commentary
Source: Environ Health. 2019 Apr 30;18:37. doi: 10.1186/s12940-019-0475-5 (PMC6489338; doi:10.1186/s12940-019-0475-5)
Supplement: Supplementary file 2 — Cumulative relative health indicator formulas, adapted with modifications from Alfredo et al. (2017). (DOCX 16 kb) [file 12940_2019_475_MOESM2_ESM.docx]

**Additional File 2**

*Cumulative Relative Health Indicator Formulas*

(adapted with modifications from Alfredo [8]).

1. Non-cancer Relative Health Indicator score = Σ ([Exposure] x [Non-cancer Severity Factor] x [Incidence of non-cancer effect] / [Non-cancer health benchmark])
2. Cancer Relative Health Indicator score = Σ ([Exposure] x [Cancer Severity Factor] / [Contaminant concentration corresponding to 10^-6^ lifetime cancer risk])

Where:

- Exposure is the arithmetic mean for the range of concentrations reported for an individual contaminant in a specific water system.
- Severity factors are equivalent to disability weights used for Global Burden of Disease studies where disease impacts range between 0 (perfect health) to 1 (mortality).
- Incidence factor represents the postulated percentage of the population likely to experience adverse health effects in response to a given concentration of contaminant. Seidel [7] and Alfredo [8] used a a fixed incidence factor of 1%, corresponding to a mathematical parameter of 0.01 in the non-cancer Relative Health Indicator formula.
- Cumulative Relative Health Indicator score is the sum of cancer and non-cancer scores

In the original study published by the Water Research Foundation, Relative Health Indicator scores are expressed with a log-based metric in the E-6 to E-4 range [7]. For ease of visualizing the analysis, we converted the scores to a simple numeric scale by multiplying the relative health indicator scores by 10^6^.
